# HMGB1 interacts with XPA to facilitate the processing of DNA interstrand crosslinks in human cells

**DOI:** 10.1093/nar/gkv1183

**Published:** 2015-11-17

**Authors:** Anirban Mukherjee, Karen M. Vasquez

**Affiliations:** Division of Pharmacology and Toxicology, College of Pharmacy, The University of Texas at Austin, Dell Pediatric Research Institute, 1400 Barbara Jordan Boulevard, Austin, TX 78723, USA

## Abstract

Many effective agents used in cancer chemotherapy cause DNA interstrand crosslinks (ICLs), which covalently link both strands of the double helix together resulting in cytotoxicity. ICLs are thought to be processed by proteins from a variety of DNA repair pathways; however, a clear understanding of ICL recognition and repair processing in human cells is lacking. Previously, we found that the high mobility group box 1 (HMGB1) protein bound to triplex-directed psoralen ICLs (TFO-ICLs) *in vitro*, cooperatively with NER damage recognition proteins, promoted removal of UVC-induced lesions and facilitated error-free repair of TFO-ICLs in mouse fibroblasts. Here, we demonstrate that HMGB1 recognizes TFO-ICLs in human cells, and its depletion increases ICL-induced mutagenesis in human cells without altering the mutation spectra. In contrast, HMGB1 depletion in XPA-deficient human cells significantly altered the ICL-induced mutation spectrum from predominantly T→A to T→G transversions. Moreover, the recruitment of XPA and HMGB1 to the ICLs is co-dependent. Finally, we show that HMGB1 specifically introduces negative supercoils in ICL-containing plasmids in HeLa cell extracts. Taken together, our data suggest that in human cells, HMGB1 functions in association with XPA on ICLs and facilitates the formation of a favorable architectural environment for ICL repair processing.

## INTRODUCTION

DNA interstrand crosslinking agents covalently link both strands in duplex DNA, creating DNA interstrand crosslinks (ICLs), which present formidable barriers for DNA metabolic processes such as DNA replication and transcription. ICL-inducing agents are cytotoxic and are often repaired in an error-generating fashion, resulting in chemoresistance and genomic instability in mammalian cells [reviewed in (1)]. Because of their cytotoxicity, crosslinking agents such as platinum compounds, mitomycin C, nitrogen mustards and psoralens are used clinically for the treatment of cancer (2). However, treatment with ICL-inducing agents can result in drug resistance and/or the development of secondary malignancies (3,4). Therefore, a better understanding of the mechanisms involved in ICL repair in human cells is warranted and may lead to the identification of novel pharmacological targets to improve the efficacy of cancer chemotherapy. While the repair of ICLs has been well characterized in bacteria (5,6), yeast (7) and in *Xenopus-*based *in vitro* systems (8,9), their repair in mammals is not clearly understood. Several DNA repair pathways have been implicated in the processing of ICLs in mammalian cells, such as nucleotide excision repair (NER), transcription-coupled NER, base excision repair (BER), mismatch repair (MMR), homologous recombination repair (HR) and proteins involved in the Fanconi anemia pathway (FA) [reviewed in (10,11)].

ICLs can be directed to specific sites by covalent conjugation of the crosslink-forming agent to a triplex-forming oligonucleotide (TFO), which binds to duplex DNA in a sequence-specific fashion via Hoogsteen hydrogen bonding (12–15). Such TFO-directed ICLs have been extensively used to study the repair of ICLs [reviewed in (16)]. For example, it has been demonstrated by our group and others that TFO-directed ICLs are substrates for NER (15,17,18) and processing of such lesions can occur in an error-generating fashion. NER damage recognition protein complexes, XPC-RAD23B and XPA-RPA interact with TFO-directed ICLs (15,19), and the NER structure-specific nuclease, XPF-ERCC1, has also been implicated in TFO-directed ICL processing in mammalian cells (20). In addition to NER damage recognition proteins, helix-distorting lesions, such as psoralen ICLs are attractive targets for architectural proteins. For example, the high mobility group box 1 (HMGB1) protein, a highly abundant non-histone architectural protein, binds to structurally distorted DNA, including TFO-directed psoralen ICLs, with higher affinity than canonical double-stranded DNA [reviewed in (21)]. HMGB1 has two box domains, an N-terminal BoxA domain, which binds to DNA in a non-sequence specific manner (22) and a BoxB domain that bends DNA (23). An acidic C-terminal tail stabilizes the interaction of the two box domains (24). In addition to a role in DNA repair, HMGB1 serves as an activator for protein TP53 (25), and when secreted from cells plays an important role in inflammation and tumor progression (26,27). In the context of DNA repair, HMGB1 has been shown to interact with proteins from the NER, BER, MMR and V(D)J recombination pathways [reviewed in (21)]. We have previously demonstrated that HMGB1 recognized TFO-directed ICLs specifically and with high affinity *in vitro* in a positive cooperative fashion with the NER proteins XPA, RPA and XPC-RAD23B (28,29). Further, we have shown that HMGB1 enhanced the error-free repair of psoralen ICLs in mouse embryonic fibroblasts (MEFs), and promoted cell survival (30).

In this study, we explored the function of HMGB1 in the recognition and processing of TFO-directed ICLs in human cells and whether its function was dependent on TP53. We found that HMGB1 was enriched at TFO-directed ICLs (relative to undamaged DNA) in human cells. Using *supF-*based mutagenesis assays we found that ICLs were equally mutagenic in TP53-proficient and -deficient human cells with comparable mutation spectra in the presence or absence of HMGB1, suggesting a TP53-independent effect of HMGB1 in processing TFO-directed ICLs. We also assessed whether HMGB1 was associated with the NER damage recognition/processing proteins in the context of the TFO-directed ICLs. Using mutagenesis assays we demonstrated that in XPA-deficient human cells, depletion of HMGB1 had no additional effect on the ICL-induced mutation frequency. However, sequencing of the ICL-induced mutants revealed a significant alteration in the mutation spectrum from predominantly T→A to T→G transversions. Further, we found that HMGB1 promoted damage-specific recruitment of XPA to ICLs in human cells. Finally, we demonstrated that HMGB1 preferentially introduced negative supercoils in ICL-containing plasmids in HeLa cell extracts. Thus, we provide the first evidence to suggest a function of HMGB1 in NER-associated ICL repair in human cells. We conclude that HMGB1 binds to the TFO-directed ICLs in human cells and supports NER processing of the TFO-directed ICLs by facilitating the recruitment of the NER recognition/verification factor XPA to the damaged sites while facilitating the formation a favorable DNA topology for lesion repair.

## MATERIALS AND METHODS

### Cell culture and siRNA transfection

Human osteosarcoma (U2OS) and cervical cancer (HeLa) cells were grown in Dulbecco's modified Eagle's medium media supplemented with 10% Fetal Bovine Serum (FBS) and 1% penicillin/streptomycin at 37°C with 5% CO_2_. The XPA^−/−^ (XP12RO) cells were grown in RPMI media supplemented with 10% FBS and 1% penicillin/streptomycin. The XPA^+/+^ (Clone 12) cells were grown in similar media under 1% G418 (Life Technologies) selection. HMGB1 depletion was achieved by reverse transfection of 400 000 cells with SmartPool siGENOME HMGB1 siRNA at 20 nM final concentration (Thermo Scientific) with Lipofectamine RNAiMAX (Invitrogen) transfection reagent to a final volume of 2.5 ml in Optimem (Life Technologies) in a 60 mm dish as per manufacturer's recommendation. Non-target siGENOME siRNA (Thermo Scientific) was used as a control. Twenty-four hours later, cells were treated (forward transfection) with a second round of siRNA (Supplementary Figure S1). To detect the efficiency of siRNA-mediated depletion of HMGB1, cells were collected at 24 and 96 h (4 days) after the first transfection and 20 μg of whole cell lysate was resolved by 10% sodium dodecyl sulphate-polyacrylamide gel electrophoresis and transferred onto a PVDF membrane. Anti-HMGB1 rabbit polyclonal antibody (Abcam) and anti-β-actin rabbit polyclonal antibody (Abcam) were used at 1:1000 dilution and proteins were detected using ECL Prime detection kit (GE Healthcare) by a BIO-RAD Chemidoc imaging system.

### TFO-directed DNA interstrand crosslink formation

**Figure 1.**
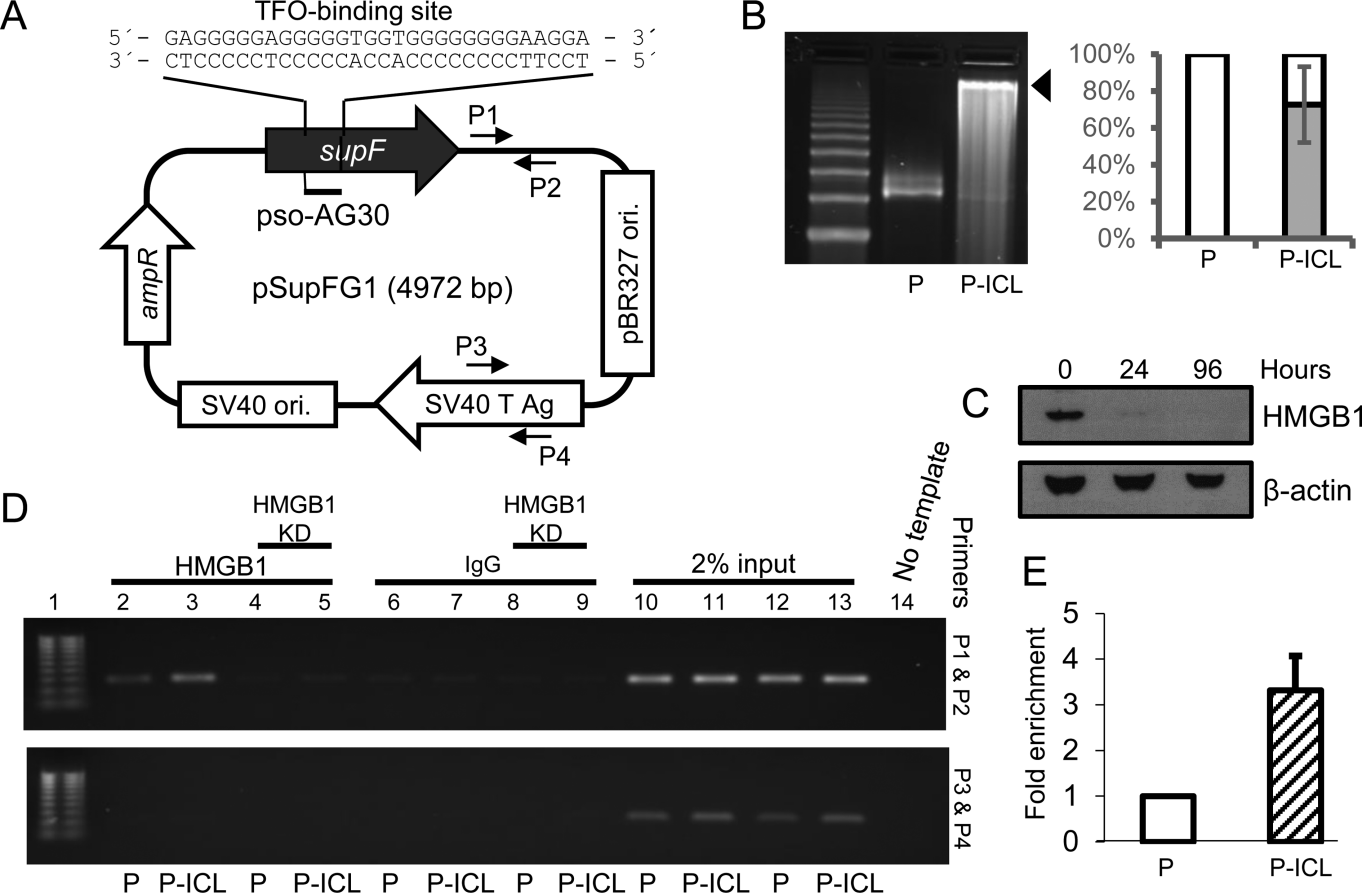
HMGB1 binds TFO-directed ICLs in human cells. (**A**) Schematic representation of the pSupFG1 plasmid containing the TFO pAG30-binding site within the *supF* gene. P1 and P2 indicate the locations of the forward and reverse primers proximal to the ICL; P3 and P4 indicate the locations of the control distal primer set. (**B**) Left panel, plasmid pSupFG1 (P) alone or pSupFG1 incubated with pAG30 and then irradiated with UVA at 1.8 J/cm^2^ (P-ICL) were resolved by 1% alkaline agarose gel electrophoresis to determine the efficiency of ICL formation (indicated by the black arrow). Right panel, quantification of three gels indicated that an average of ∼70% of the plasmid substrates contained TFO-directed ICLs. Error bars represent ± standard deviation (SD). (**C**) Western blot demonstrating the efficiency of HMGB1 depletion at 24 and 96 h (4 days) after siRNA treatment in U2OS cells. (**D**) PCR analysis of ChIP assays from U2OS cells demonstrated an enrichment of HMGB1 at the TFO-directed psoralen ICL region with proximal primers P1 and P2 (top panel) and distal control primers P3 and P4 (bottom panel). Lane 1, 100 bp ladder; lane 2, control plasmid (P); lane 3, ICL-containing plasmid (P-ICL). Lanes 4 and 5 are similar to lanes 2 and 3, respectively, but following siRNA-mediated depletion of HMGB1 from U2OS cells. Lanes 6, 7, 8 and 9 are IgG controls for lanes 2, 3, 4 and 5, respectively. Lanes 10–13 represent 2% input, lane 14 is a no template PCR control. (**E**) Quantification of PCR amplification from C indicated greater than three-fold enrichment of HMGB1 on the ICL-containing plasmid (P-ICL) compared to the control plasmid (P), *n* = 4, error bar represents ± SD.

TFO, AG30 containing a covalent 5′ HMT-psoralen (pso-AG30) was synthesized and HPLC purified by Midland Certified Reagent Co (Midland, TX, USA) as previously described (31). For mutagenesis assays, equimolar amounts of pso-AG30 and plasmid pSupFG1, harboring a TFO-binding site within the *supF* region, were incubated in an amber tube with triplex binding buffer (50% glycerol, 10 mM Tris (pH 7.6), 10 mM MgCl_2_) at 37°C overnight followed by 1.8 J/cm^2^ UVA (365 nm) irradiation on ice under a Mylar filter. The TFO-binding site is located within the *supF* gene in the plasmid adjacent to a 5′-AT-3′ psoralen crosslinking site at the triplex–duplex junction (Figure 1A). To confirm and quantify triplex-directed ICL formation, plasmids were linearized by EcoRI digestion, heat denatured and resolved on a 1% alkaline agarose gel, stained with SYBR gold and visualized using a BIORAD Chemidoc imaging system (Figure 1B). Densitometric quantification of band intensities was performed using ImageQuant software (GE Healthcare Life Sciences).

### Chromatin immunoprecipitation assay

Binding of HMGB1 and XPA to the plasmid with or without a site-specific TFO-directed psoralen ICL in human cells were assessed by using the Simple ChIP Enzymatic Chromatin IP kit (Cell Signaling Inc.) following a previously described protocol (32). Briefly, U2OS cells were treated with HMGB1 siRNA twice (Supplementary Figure S1). The first transfection was performed using RNAiMAX and 24 h later, the siRNA and the plasmids were transfected using GenePORTER (Genlantis). Cells were harvested 24 h following plasmid transfection and chromatin immunoprecipitation was performed as per the manufacturer's suggestion. The cells were treated with 37% fresh formaldehyde to a final concentration of 1% at room temperature for 10 min. Formaldehyde crosslinking was quenched using 125 mM chilled glycine for 5 min at room temperature and cells were collected by scraping. Pellets were treated with micrococcal nuclease and nuclei were pelleted, and then pulse sonicated on ice for 4 min total using 50 amplitude (Episonic) to generate ∼800–1200 bp DNA fragments. Lysates were then incubated with anti-HMGB1 rabbit polyclonal antibody (Abcam), rabbit polyclonal immunoglobulin G (IgG) antibody (Cell Signaling), rabbit monoclonal histone H3 antibody (Cell Signaling), anti-XPA rabbit polyclonal antibody (Abcam) overnight at 4°C with rotation followed by incubation with protein G magnetic beads for 4 h at 4°C. The formaldehyde crosslinking was reversed by incubating the samples with 5 M NaCl and Proteinase K at 65°C for 2 h. Samples were purified using the Wizard gel and polymerase chain reaction (PCR) clean up kits (Promega) and subjected to PCR using GoTaq Green master mix (Promega) and amplified for 30 cycles in a BIORAD thermal cycler by subjecting the samples to 30 s at 95°C, 45 s at 56°C, 30 s at 72°C and a final 5 min extension time at 72°C. The primers used for PCR amplification were: Primer P1, 5′-gcc ccc ctg acg agc atc ac; Primer P2, 5′-tag tta ccg gat aag gcg cag cgg; Primer P3, 5′-aat acc gcg cca cat agc ag; and Primer P4, 5′-agt att caa cat ttc cgt gtc gcc. The PCR products were resolved on a 1% agarose gel and visualized by ethidium bromide staining using a BIORAD Chemidoc imaging system. The band intensities were determined using ImageQuant (GE Healthcare Life Sciences). The relative enrichment of PCR products was measured by subtracting the background and normalized twice, first against input samples, followed by untreated control plasmids. Statistical analysis was performed using Student's *t*-tests.

### Mutagenesis assay

To determine the effect of HMGB1 in processing TFO-directed ICLs in human cells, 2 μg of plasmid pSupFG1 with or without TFO-directed ICLs were transfected into HMGB1-depleted U2OS, HeLa and XPA-proficient or -deficient cells (Supplementary Figure S1). Cells were reverse transfected with HMGB1 siRNA using RNAiMAX. Twenty-four hours later, a second round of HMGB1 siRNA transfection was performed in association with plasmids using GenePORTER (Invitrogen) transfection reagent per the manufacturer's suggestions. Mutagenesis assays were performed as we have described (31). In brief, cells were harvested 48 h after the plasmid transfection and plasmid DNA was isolated using QIAprep Spin Miniprep kit (QIAGEN, Inc.). To remove unreplicated plasmids from the analysis, plasmids were treated with DpnI restriction endonuclease for 1 h at 37°C followed by phenol/chloroform/isoamyl alcohol extraction and ethanol precipitation with 0.3 M sodium acetate (pH 5.2). Subsequently the plasmids were re-suspended in 10 μl nuclease-free water. Mutations in the *supF* gene due to processing of the TFO-directed ICLs were detected by transforming 30 μl of electro-competent *Escherichia coli* cells MBM7070 (Lucigen) with 1 μl of plasmid DNA and then plating on X-gal, IPTG and ampicillin containing agar plates for blue/white screening. A functional *supF* gene produces a blue colony, while a mutated *supF* gene produces a white colony. Mutation frequencies were calculated by dividing the number of white colonies counted by the total number of colonies counted. Three individual experiments were performed for each mutagenesis assay and ∼30 000 colonies were counted per experiment and more than 1 million colonies were counted in total. The *P*-values were calculated by the Holm-Sidak one-way analysis of variance method using Sigmaplot. Mutation spectra were determined by direct sequencing of the plasmid DNA.

### Supercoiling assays

Three hundred nanogram of pSupFG1 or TFO-directed ICL-containing pSupFG1 plasmids were incubated in HeLa cell extracts or in siRNA-mediated, HMGB1-depleted HeLa cell extracts containing DNA repair synthesis buffer (45 mM Hepes-KOH, pH 7.8; 70 mM KCl, 7.4 mM MgCl_2_; 0.9 mM DTT; 0.4 mM EDTA; 2 mM ATP), 20 μM dNTPs, 40 mM phosphocreatine, 2.5 μg creatine phosphokinase, 3.4% glycerol and 18 μg bovine serum albumin. The reaction was mixed on ice and incubated at 37°C for 1 h. The reactions were terminated by incubating with 1% SDS and 0.25 mg/ml proteinase K for 2 h at 65°C and loaded directly onto a 1% agarose gel and run for 8 h at ∼2 V/cm in 1× Tris Borate EDTA (TBE) to resolve the topoisomers. For the second dimension, to distinguish between the positive and the negative supercoils, the gels were soaked in 3 μg/ml chloroquine containing 1× TBE for 20 min, turned 90 degrees and run for another 4 h at ∼2 V/cm in chloroquine containing 1× TBE. The gels were then soaked in SYBR Gold (Invitrogen) and visualized using the BIORAD Chemidoc imaging system. The negative superhelical turns are identified as a left-handed arc and the positive supercoils are identified as a right-handed arc.

## RESULTS

### HMGB1 binds to TFO-directed psoralen interstrand crosslinks in human cells

The plasmid pSupFG1 used for these studies contains a TFO-binding site (AG30) within the mutation-reporter gene (Figure 1A) adjacent to a 5′-AT-3′ psoralen crosslinking site. Formation of the TFO-directed ICLs on pSupFG1 was confirmed via 1% alkaline agarose gel electrophoresis (Figure 1B). siRNA-mediated depletion of HMGB1 in U2OS cells was >90% for up to 96 h as assessed via western blot analysis (Figure 1C). To determine if HMGB1 interacts with the TFO-directed psoralen ICLs in human cells, we performed modified chromatin immunoprecipitation (ChIP) assays in U2OS cells (Figure 1D). PCR amplification following immunoprecipitation with HMGB1 antibodies using the ICL proximal primers (P1 and P2, Figure 1A) revealed a greater than three-fold enrichment of HMGB1 at the TFO-directed ICL site compared to untreated substrates (Figure 1D, lanes 2 and 3, upper panel; quantification is shown in Figure 1E) in U2OS cells. No such difference in enrichment was observed between the damaged and undamaged substrates in HMGB1-depleted U2OS cells, as expected (Figure 1D, lanes 4 and 5, upper panel). As an additional control, PCR amplification was performed using the distal control primers (P3 and P4, Figure 1A) and no enrichment of HMGB1 was detected at the control (undamaged) region (Figure 1D, bottom panel). These results indicate that HMGB1 is specifically recruited to TFO-directed ICLs and is the first demonstration of association of HMGB1 with TFO-directed ICLs in human cells.

### HMGB1 promotes error-free repair of TFO-directed ICLs in human cells

**Figure 2.**
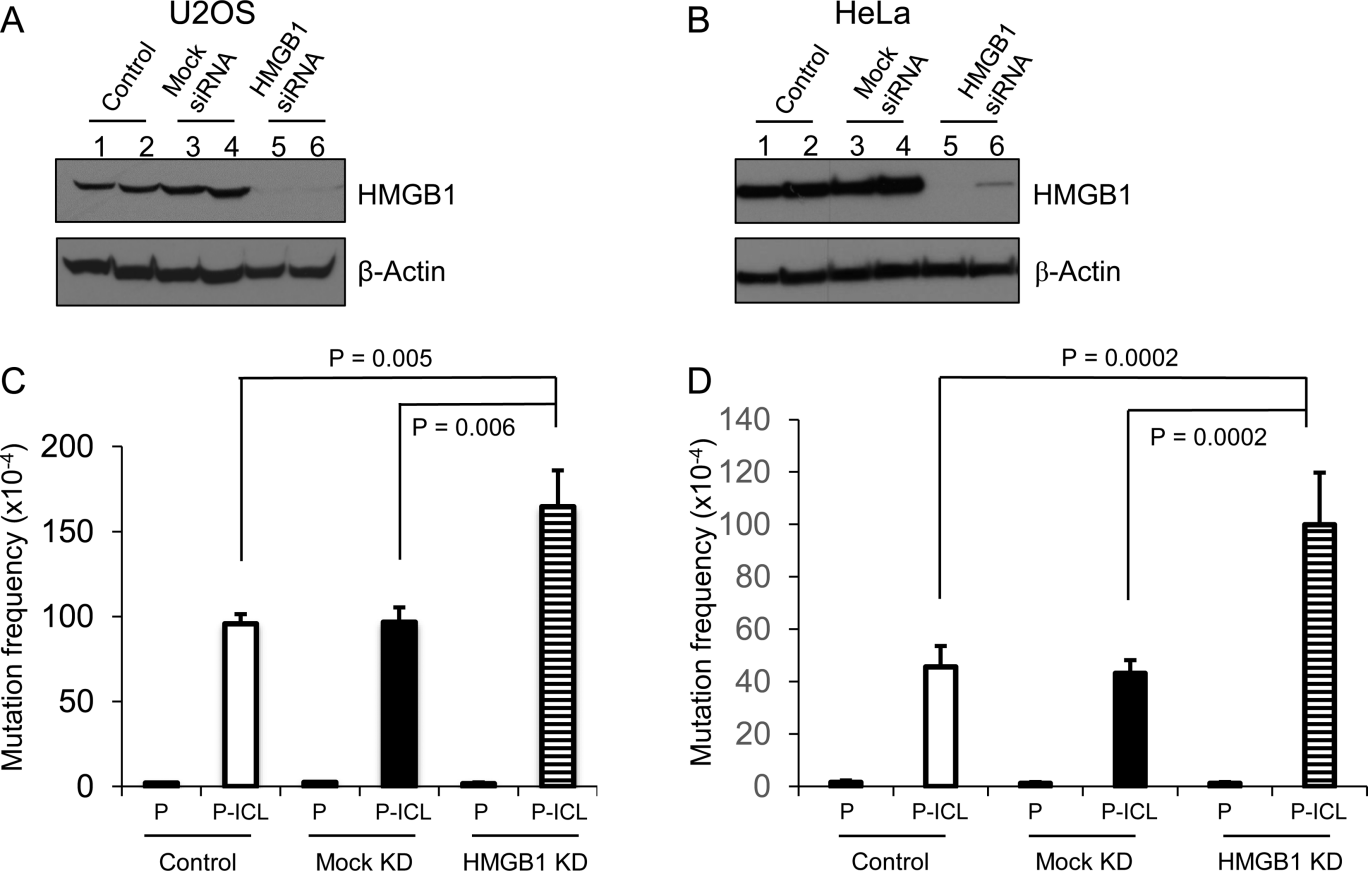
HMGB1 depletion in human cells increases psoralen ICL-induced mutagenesis. (**A** and **B**) Western blot to assess siRNA-mediated depletion of HMGB1 in U2OS (TP53 proficient) and HeLa (TP53 deficient) cells, respectively. Lanes 1 and 2, untreated control; lanes 3 and 4, mock-siRNA treated; lanes 5 and 6, HMGB1-siRNA treated. (**C** and **D**) Spontaneous (P) and ICL-induced (P-ICL) mutation frequencies in HMGB1-depleted U2OS and HeLa cells, respectively. Cells were transfected with pSupFG1 plasmid (P) or with TFO-directed psoralen-crosslinked pSupFG1 (P-ICL), as indicated under the bars. The results are an average of four independent experiments; error bars represent ± SD. *P*-values derived (from one-way ANOVA (Holm-Sidak method).

Based on our ChIP results, we tested the biological consequence of the interaction of HMGB1 with TFO-directed ICLs in human cells. Previously, we found that knockout of HMGB1 in TP53-deficient MEFs resulted in increased psoralen ICL-induced mutagenesis, suggesting a requirement for HMGB1 in the error-free processing of these lesions (30). However, the interaction of HMGB1 with TP53 has been implicated in cellular processes, such as apoptosis (33). Thus, we wanted to determine if HMGB1 was involved in the error-free processing of TFO-directed ICLs in human cells and whether this function was dependent on functional TP53. We performed ICL-induced mutagenesis assays in U2OS cells containing functional TP53, and in HeLa cells, which have non-functional TP53. HMGB1 was depleted using an siRNA approach in both U2OS and HeLa cells and the efficiency of depletion was >90%, as assessed by western blotting (Figure 2A and B, lanes 5 and 6). Processing of the TFO-directed ICLs in U2OS cells resulted in an ∼45-fold induction in ICL-induced mutagenesis when compared to the undamaged substrate (Figure 2C). In comparison, depletion of HMGB1 resulted in an ∼102-fold induction in mutagenesis (Figure 2C), revealing an approximately two-fold increase in ICL-induced mutation frequencies as a function of HMGB1 deficiency. Similar mutagenesis assays performed in HeLa cells demonstrated ∼30-fold induction in TFO-directed ICL-induced mutation frequencies over undamaged control substrates, while HMGB1 depletion resulted in ∼83-fold induction, resulting in an approximately two-fold increase in mutation frequencies similar to U2OS cells (Figure 2D). Though the induction of mutagenesis was significantly higher when HMGB1 was depleted from both U2OS and HeLa cells, no significant differences were observed in the presence or absence of functional TP53, suggesting that the involvement of HMGB1 in the error-free processing of the ICLs was independent of its association with functional TP53. DNA from 20 random mutant colonies was isolated for each experimental group and sequenced to identify the types of mutations generated at the TFO-targeted 5′-AT-3′ psoralen crosslinking site. The mutation spectra revealed primarily T→A transversions along with other point mutations (Supplementary Figure S2), consistent with our previously published results in HMGB1-proficient and deficient MEFs (30). In conclusion, HMGB1 depletion increased the overall number of mutations while processing the TFO-directed ICLs in human cells without altering the mutation spectra, suggesting a low fidelity repair processing of these lesions in the absence of HMGB1.

### HMGB1 is involved in NER-associated ICL-induced mutagenesis

**Figure 3.**
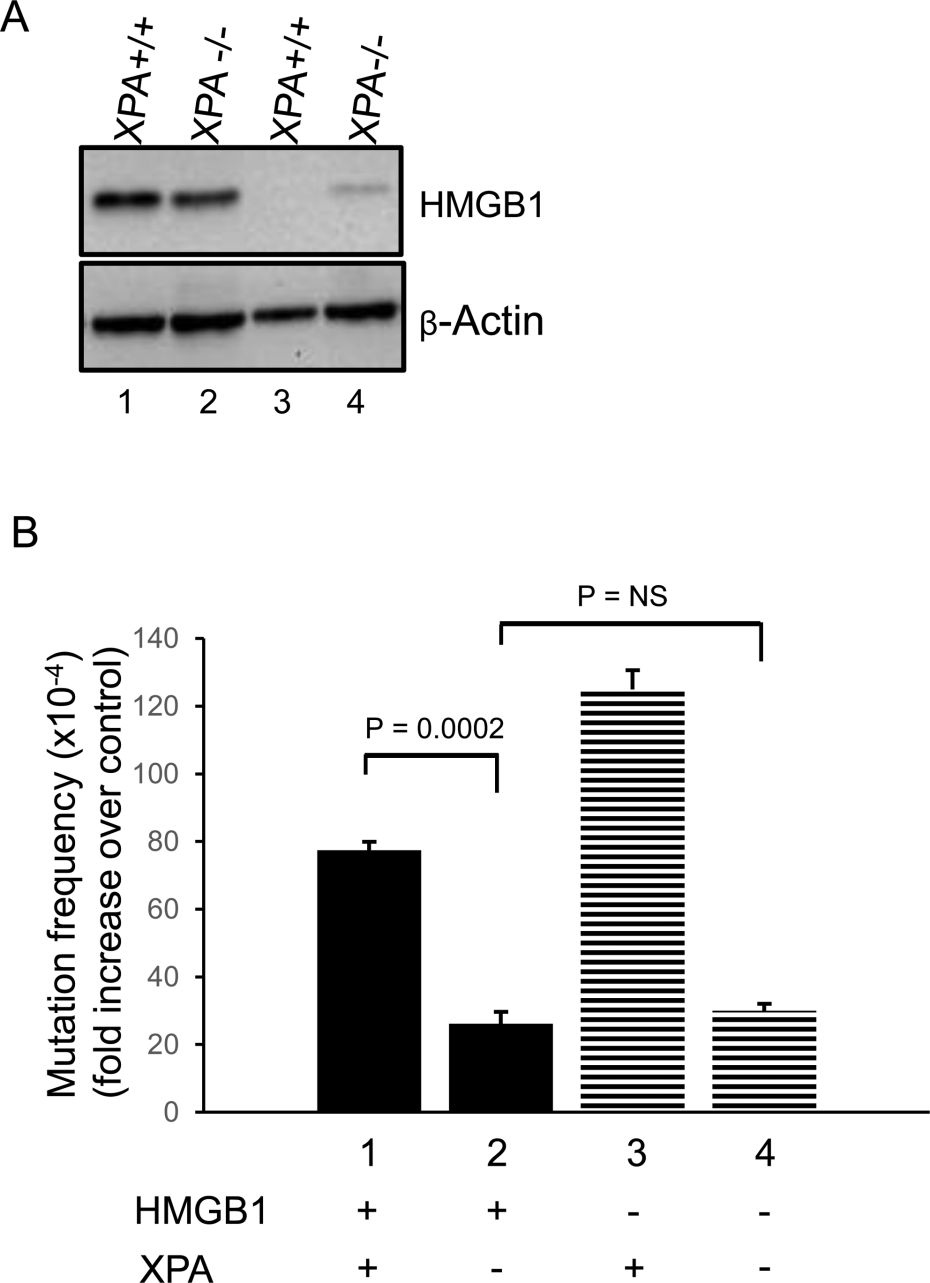
ICL-induced mutagenesis is stimulated by HMGB1 depletion and is associated with XPA in human cells. (**A**) Western blot to assess siRNA-mediated depletion of HMGB1 in XPA-proficient or XPA-deficient fibroblasts. (**B**) Fold increase in mutation frequencies induced by ICLs over spontaneous frequencies. TFO-directed ICL-induced mutation frequencies over undamaged control in XPA-proficient fibroblasts (bar 1). XPA deficiency decreases the ICL-induced mutation frequency by approximately three-fold (bar 2). HMGB1 depletion in XPA-proficient cells (panel A, lane 3) increases the ICL-induced mutation frequency (bar 3). In XPA-deficient and HMGB1-depleted cells (bar 4), the mutation frequency is similar to XPA-deficient cells (bar 2). These results are an average of three independent experiments; error bars indicate ± SD. Statistical significance determined by one-way ANNOVA (Holm-Sidak method).

Previously, we found that HMGB1 interacts with NER proteins on TFO-directed psoralen ICLs *in vitro* (28,29) and that the efficiency of NER-dependent UV lesion removal was reduced in HMGB1-deficient MEFs, implicating HMGB1 as an NER co-factor (30). Therefore, we wanted to advance our understanding of the association of HMGB1 with NER proteins in the processing of TFO-directed ICLs in human cells. To address this, we performed mutagenesis assays in XPA-deficient (XP12RO) and isogenic XPA-proficient human cells (Clone 12) (34). HMGB1 depletion in the XPA-proficient and -deficient cell lines was >90%, as assessed by western blot analyses (Figure 3A, lanes 3 and 4). For purposes of comparison, the results from the mutagenesis assays are plotted as the fold increase in ICL-induced mutation frequencies over untreated control (P-ICL/P) (Figure 3B). In the XPA-proficient Clone 12 cells, the TFO-directed psoralen ICLs induced mutagenesis ∼75-fold over the background levels (Figure 3B, bar 1). In contrast, the ICL-induced mutagenesis was significantly reduced (to ∼25-fold over background) in the XPA-deficient XP12RO cells (Figure 3B, bar 2). When similar mutagenesis assays were performed after depleting HMGB1 in the XPA-proficient cells, the mutation frequency was ∼125-fold over the background spontaneous mutation frequency (Figure 3B, bar 3), indicating an approximately two-fold increase in ICL-induced mutagenesis over that seen in wild-type cells, consistent with our results from U2OS and HeLa cells. However, depletion of HMGB1 in XPA-deficient cells did not result in the characteristic increase in ICL-induced mutagenesis (Figure 3, bar 4). Rather, the mutation frequencies from XPA-deficient cells versus HMGB1-depleted, XPA-deficient cells were similar (∼25 and ∼30-fold, respectively; Figure 3B, compare bars 2 and 4). These results are consistent with a previous report demonstrating a role for XPA in TFO-directed ICL processing (17), and provide additional evidence to suggest that HMGB1 and XPA function in a similar pathway in the processing of TFO-directed ICLs in human cells.

### HMGB1 depletion in XPA-deficient fibroblasts significantly alters the pattern of ICL-induced transversion events

ICL-induced mutants generated in the human cell lines were sequenced to characterize the mutation spectra. Sequencing results from mutants generated in XPA-proficient and XPA-deficient human cells revealed ∼75% base substitutions at the ICL-targeted site, with the remaining mutants containing small deletions within and surrounding the ICL-targeted site (Table 1 and Supplementary Figure S3). HMGB1 depletion in these XPA-proficient and XPA-deficient cells did not significantly alter the ICL-induced base substitutions. However, HMGB1 depletion in both the XPA-proficient and XPA-deficient cells resulted in an increase in small deletions (from ∼25 to ∼50%; Table 1). We further analyzed the types of base substitutions and observed a shift in the predominant transversion event (Table 2). The T→A transversions in XPA-proficient cells accounted for ∼45% of the total mutations, which was significantly reduced compared to ∼15% in the XPA-deficient, HMGB1-depleted cells (*P* = 0.034, Z-test). On the other hand, the major transversion event observed in XPA-deficient cells when HMGB1 was depleted was a T→G transversion at the ICL-targeted site, which accounted for ∼45% of all mutations indicating a significant increase from ∼10% (*P* = 0.016, Z-test) as observed in the wild-type cells (Table 2). In summary, deficiency of HMGB1 and XPA together results in a significant alteration in the type of transversion event, consistent with mutagenic processing of TFO-directed ICLs in human cells.

**Table 1.** ICL-induced mutation spectra in XPA-proficient and -deficient human cells in the presence or absence of HMGB1

	Base substitution	Insertion	Deletion
XPA+/+	75% (15/20)	0% (0/20)	25% (5/20)
XPA−/−	75% (15/20)	0% (0/20)	25% (5/20)
HMGB1 KD			
XPA+/+	75% (15/20)	0% (0/20)	45% (9/20)*
XPA−/−	70% (14/20)	5% (1/20)	50% (10/20)*

*Clones containing more than one mutation.

*n* = 20; mutants sequenced from three independent experiments.

The percentages shown have been calculated as mutations per plasmid sequenced.

**Table 2.** ICL-induced transversions in XPA-proficient and -deficient human cells in the presence or absence of HMGB1

Transversions	XPA+/+	XPA−/−	XPA+/+ (HMGB1-depleted)	XPA−/− (HMGB1-depleted)
			HMGB1 depleted	
T→A	45%* (9/20)	**30%** (6/20)	**35%** (7/20)	15% (3/20)
T→G	10% (2/20)	15% (3/20)	15% (3/20)	**45%** (9/20)

*Bold font highlights the predominant transversion event.

*n* = 20; mutants sequenced from three independent experiments.

The percentages shown have been calculated as mutations per plasmid sequenced.

### The recruitment of HMGB1 and XPA to ICLs is interdependent in human cells

**Figure 4.**
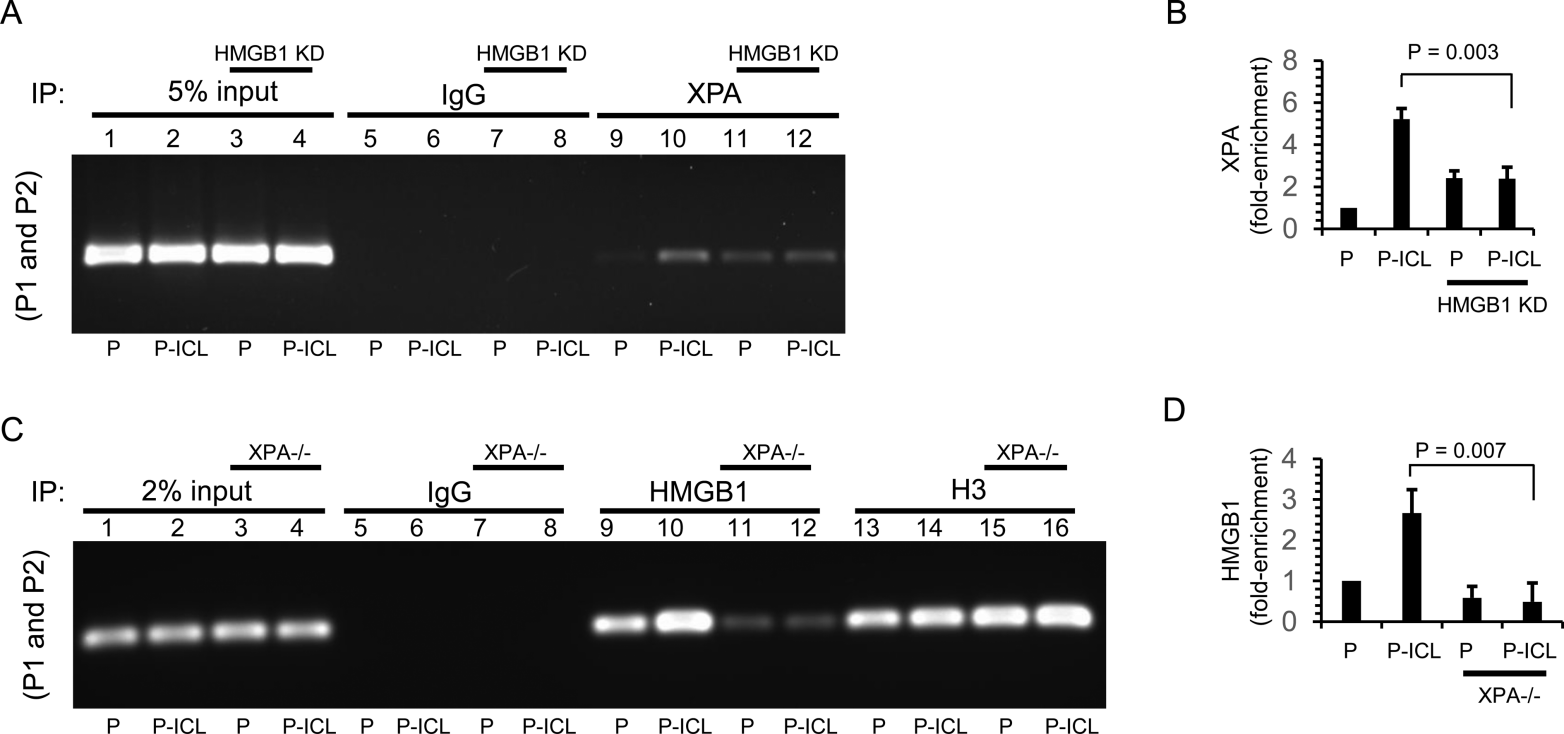
The association of XPA and HMGB1 on psoralen ICLs is interdependent in human cells. (**A**) PCR analysis from ChIP experiments on control and HMGB1-depleted (HMGB1 KD) U2OS cells transfected with plasmid pSupFG1 (P) (lanes 1, 3, 5, 7, 9 and 11) or psoralen-crosslinked pSupFG1 (P-ICL) (lanes 2, 4, 6, 8, 10 and 12) and immunoprecipitated with indicated antibodies (IgG and HMGB1). PCR analysis of ChIP samples using proximal primers P1 and P2 is shown. (**B**) Quantification of PCR products from A. (**C**) ChIP experiments with P (lanes 1, 3, 5, 7, 9, 11, 13 and 15) or P-ICL (lanes 2, 4, 6, 8, 10, 12, 14 and 16) transfected into XPA-proficient (XPA+/+) and XPA-deficient (XPA−/−) cells and immunoprecipitated with indicated antibodies (IgG, HMGB1, H3) followed by PCR amplification with the P1 and P2 primers. (**D**) Quantification of PCR products from C. Error bars represent ± SD, *n* = 3, statistical significance determined using Student's *t*-test.

HMGB1 is an architectural protein that interacts with TFO-directed ICLs with high affinity (28). HMGB1 interacts with the NER damage recognition complexes XPA-RPA and XPC-RAD23B on TFO-directed ICLs *in vitro* (29) and physically interacts with XPA in the absence of damaged DNA (29). Thus, we performed ChIP assays in U2OS cells on ICL-containing plasmids using antibodies against XPA to determine whether recruitment of these proteins is modulated by HMGB1 in human cells. PCR amplification of the immunoprecipitated DNA fragments isolated from U2OS cells 24 h post-transfection with the ICL-containing plasmid clearly demonstrated an enrichment of XPA (approximately five-fold) at the TFO-directed ICL-containing region relative to the undamaged control (Figure 4A, compare lanes 9 and 10; quantified in Figure 4B), suggesting DNA damage-specific recruitment of XPA. Strikingly, when HMGB1 was depleted in the U2OS cells we observed two differences: (i) XPA was enriched at the undamaged control region ∼2.5-fold (Figure 4A, compare lanes 9 and 11); and (ii) the DNA damage-specific enrichment of XPA was significantly reduced (Figure 4A, compare enrichment between lanes 9 and 10 to that with lanes 11 and 12; quantified in Figure 4B), suggesting an interaction of XPA and HMGB1 on the ICLs in human cells. This is a novel finding demonstrating an association of XPA and HMGB1 in human cells on TFO-directed ICLs.

Conversely, to determine if XPA was required for the recruitment of HMGB1 to the ICLs we performed ChIP assays in the XPA-proficient and XPA-deficient fibroblasts using HMGB1 antibodies. PCR amplification of the immunoprecipitated fragments demonstrated an enrichment of HMGB1 on the ICL-containing region in XPA-proficient cells, but this association was significantly decreased (*P* = 0.007) in the XPA-deficient cells (Figure 4C, compare lanes 9 and 10 to lanes 11 and 12; quantified in Figure 4D). These data indicate that recruitment of HMGB1 to the ICL-damaged DNA is dependent on XPA, which is a novel finding. Taken together, these findings indicate that the recruitment of XPA to TFO-directed ICLs is modulated, at least in part, by HMGB1 in human cells, and vice versa.

### HMGB1 specifically introduces negative supercoils in ICL-containing plasmids in HeLa nuclear extracts

**Figure 5.**
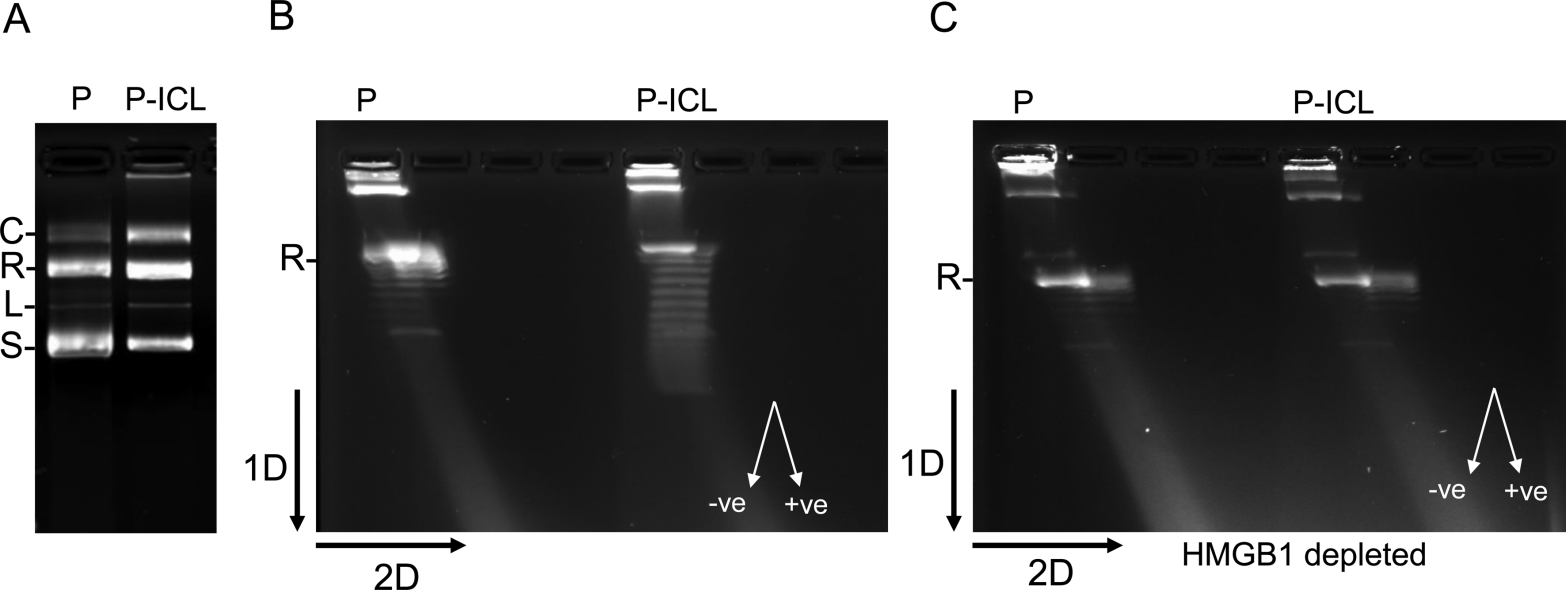
HMGB1 facilitates negative supercoiling in the psoralen crosslinked plasmid in HeLa cell extracts. (**A**) Plasmid pSupFG1 (P) and the ICL-containing plasmid (P-ICL) subjected to agarose gel electrophoresis: C, concatemers; R, nicked relaxed; L, linear; and S, supercoiled species. (**B**) Two-dimensional agarose gel electrophoresis resolving the thermal distributions of the topoisomers after 1 h incubation of P or P-ICL in HeLa extracts containing DNA repair synthesis buffer. The black arrows indicate the direction of the first and second dimensions of electrophoresis. The direction of left-handed negative and right-handed positive supercoils in the gel are indicated by white arrows. (**C**) Two-dimensional agarose gel electrophoresis similar to B, but in siRNA-mediated HMGB1-depleted HeLa extracts. All experiments were performed at least three times. The doublets (double–images) in the figure are positive and negative topoisomers of the same supercoil count.

As an architectural protein, HMGB1 can modulate the topology of DNA *in vitro* by introducing negative supercoils (35) and by stabilizing DNA loops in complex nucleoprotein structures (36). Further, architectural parameters of the DNA have been previously demonstrated to be associated with NER processing of UVC-induced lesions (37,38). Thus, due to the preferred binding of HMGB1 to TFO-directed ICLs, we wanted to test whether HMGB1 modulates the topology of the TFO-directed ICL-containing plasmids relative to the undamaged control plasmids in human cell-free extracts. To test this, we incubated the control plasmid or the ICL-containing plasmid (Figure 5A) under DNA repair synthesis conditions in either HeLa cell-free extracts or siRNA-mediated, HMGB1-depleted HeLa cell-free extracts. Subsequently, we measured the topological alterations on the plasmids in the presence or absence of HMGB1. We resolved the resulting topoisomers by two-dimensional (2D) agarose gel electrophoresis (Figure 5B). Analysis of the thermal distribution of the topoisomers demonstrated a distinct difference between the two substrates. We observed an induction of negative supercoiling specifically on the ICL-containing plasmids (Figure 5B) in HeLa cell-free extracts. In comparison, in HMGB1-depleted HeLa cell-free extracts no such induction of negative supercoiling was observed on the ICL-containing plasmids (Figure 5C). Both the control plasmids and the ICL-containing plasmids showed similar topological distributions when incubated in HMGB1-depleted HeLa extract (Figure 5C). These data indicate that HMGB1 can impose topological modifications specifically on the ICL-damaged substrates in HeLa extracts by introducing negative supercoils in the DNA. Such topological modifications have been associated with NER processing of UVC-induced DNA lesions (37,38) and thereby suggest that HMGB1 may play an important architectural role in promoting the repair processing of TFO-directed ICLs in human cells.

## DISCUSSION

ICLs can be processed in a replication-independent, NER-dependent fashion in mammalian cells when encountered in G1 or resting cells (39). We have previously demonstrated that the highly abundant architectural protein HMGB1, binds to TFO-directed psoralen ICLs *in vitro* with high affinity and specificity. Further, we found that the NER distortion/damage recognition proteins XPC-RAD23B and XPA-RPA, bind to TFO-directed psoralen ICLs *in vitro* (15,19), and interact in a positive cooperative fashion in association with HMGB1 on these lesions (28,29). Moreover, we have demonstrated that the lack of HMGB1 increased DNA damage-induced mutagenesis and reduced UV-induced adduct removal in MEFs (30). Collectively, these results suggest that HMGB1 is involved in an NER-associated processing of TFO-directed ICLs.

In this study, we found that siRNA-mediated depletion of HMGB1 did not significantly affect the cell cycle in U2OS cells (Supplementary Figure S4) consistent with our previously published results in MEFs (30). While HMGB1 is known to interact with TP53, we did not detect any significant differences in ICL-induced mutation frequencies or spectra in the presence (in U2OS cells) or absence (in HeLa and Saos-2 cells, see Supplementary Figure 5) of functional TP53 in human cells. The predominant mutation was T→A base substitutions in the cells (Supplementary Figure S2), consistent with the signature mutation of psoralen. These data indicate that in the absence of HMGB1, ICL processing is more error-prone, suggesting an overall decrease in the fidelity of ICL repair processing in human cells.

To determine the extent to which HMGB1 depletion affected NER processing of the TFO-directed ICLs, we depleted HMGB1 in XPA-deficient and XPA-proficient human fibroblasts and performed mutagenesis assays. XPA is a core NER DNA damage recognition protein and is essential for functional NER (40). Though there was a significant difference in the ICL-induced mutation frequencies, the overall mutation spectra did not change significantly in the XPA-proficeint, HMGB1-depleted cells compared to wild-type cells. We observed a small increase in ICL-induced deletions in the absence of HMGB1, which was independent of the XPA status of the cells (Table 1 and Supplementary Figure S3). Strikingly, the absence of both XPA and HMGB1 resulted in a significant shift of the major ICL-induced mutation from T→A to T→G transversions. The T→A or T→G transversions are likely the result of translesion synthesis (TLS) bypass by specialized polymerases (41). In addition, the recruitment of XPA to non-damaged sites was significantly increased in HMGB1-depleted cells, which suggests that HMGB1 may facilitate XPA damage recognition specificity. The ICL-induced mutation frequencies and spectra indicate that in the absence of HMGB1 and XPA, the processing of TFO-directed psoralen ICLs in human cells is low fidelity in nature, and these proteins may play a role in polymerase choice.

We have previously demonstrated a cooperative interaction between HMGB1 and RPA (28) and XPC-RAD23B in binding TFO-directed ICLs *in vitro* (29). We wanted to further our understanding of the nature of these interactions in human cells, where the structure-function relationship is complex due to the three dimensional organization of the eukaryotic genome. A central component of DNA damage recognition in the NER mechanism is the extent of structural distortion, which correlates with the extent of repair processing (42). The global genome damage/distortion recognition complex XPC-RAD23B causes significant bending of the DNA backbone upon binding to DNA adducts and such architectural modifications reduce the Tm value of the adducted duplex DNA (43), making it energetically favorable to process these lesions. The XPA–RPA complex, which is thought to facilitate assembly of the pre-initiation complex during NER processing of damaged DNA, has been demonstrated to bind structural alterations induced by TFO-directed ICLs with high affinity (15). Therefore it is conceivable that the *in vivo* architecture of the DNA and additional architectural proteins, such as HMGB1, may play a supporting role in lesion repair by increasing the helical flexibility to facilitate the processing of complex DNA lesions. HMGB1 is ubiquitous and highly abundant in adult human non-replicating cells, where GG-NER and TC-NER may be the choice of DNA adduct removal. For example, metaphase spreads prepared from HMGB1 knockout mouse cells are characterized by aneuploidy, tri-radial structure formation and ring-like structures (44), suggesting an important role of HMGB1 in genome maintenance. A potential outcome of HMGB1 depletion appears to be inefficient (but not total loss of) lesion processing by NER. This explanation is consistent with the delayed removal of UVC-induced lesions in HMGB1 knockout MEFs (30). On the other hand, to our surprise, the ChIP enrichment patterns indicated that the recruitment of HMGB1 to DNA is dependent on the presence of XPA, even in the absence of DNA damage. One possible explanation could be that XPA and HMGB1 are acting as a part of a complex that is associated with the DNA. It has been previously demonstrated by others that the NER factors can promote chromatin remodeling and can be recruited to inducible promoters in the absence of DNA damage (45,46). Consistent with this idea, we have previously demonstrated that HMGB1 and XPA interact in the absence of DNA, suggesting close proximity of these proteins, possibly as a part of a pre-existing complex (29).

Consistent with the architectural role of HMGB1, we found that HMGB1 induced negative supercoiling in the ICL-containing plasmids in HeLa cell extracts (Figure 5B). Such architectural modification was absent in HMGB1-depleted HeLa extracts, suggesting a damage-specific architectural modification of DNA that can be attributed to HMGB1. Negative supercoiling of the DNA has been shown to facilitate the removal of UVC-induced DNA damage in bacteria (38). Further, it has also been demonstrated in *Xenopus* egg extracts that DNA repair synthesis stimulated by UVC-induced damage in plasmids is associated with DNA supercoiling (37). We have also previously observed that HMGB1 facilitates chromatin alterations after the induction of DNA damage (30). Here we show that under DNA repair synthesis conditions, HMGB1 is capable of topologically modifying preferentially the ICL-containing substrates over non-damaged plasmids by introducing negative supercoils. Therefore, it is plausible that HMGB1 may promote a favorable architecture for NER processing of the ICLs in a cellular environment following its association with the lesion.

The role of HMGB1 in DNA damage processing has been debated and opposing theories such as ‘repair shielding’ due to less efficient recruitment of repair machinery on cisplatin-DNA adducts (47) or ‘repair enhancing’ supported by the more efficient processing of psoralen ICLs (30) have been put forward. However, in highly proliferating transformed cells, there are several other architectural proteins present. Among them are other members of the HMGB family: HMGB2, HMGB3 and HMGB4, which share high sequence similarity and in some cases functional similarities with HMGB1. Future studies to elucidate the roles of these other HMGB family members, individually or in combination, in the processing of genome destabilizing lesions and as mediators of DNA architecture are warranted.

## Supplementary Material

SUPPLEMENTARY DATA
