# Supplementary material for: HMGB1 interacts with XPA to facilitate the processing of DNA interstrand crosslinks in human cells
Source: Nucleic Acids Res. 2015 Nov 17;44(3):1151–60. doi: 10.1093/nar/gkv1183 (PMC4756816; doi:10.1093/nar/gkv1183)
Supplement: SUPPLEMENTARY DATA [file supp_44_3_1151__index.html]

HMGB1 interacts with XPA to facilitate the processing of DNA interstrand crosslinks in human cells — SUPPLEMENTARY DATA 

# HMGB1 interacts with XPA to facilitate the processing of DNA interstrand crosslinks in human cells

## SUPPLEMENTARY DATA

- SUPPLEMENTARY DATA
