# Supplementary material for: HMGB1 interacts with XPA to facilitate the processing of DNA interstrand crosslinks in human cells
Source: Nucleic Acids Res. 2015 Nov 17;44(3):1151–60. doi: 10.1093/nar/gkv1183 (PMC4756816; doi:10.1093/nar/gkv1183)
Supplement: SUPPLEMENTARY DATA [file supp_gkv1183_nar-01574-d-2015-File007.pdf]

SUPPLEMENTARY MATERIALS

SUPPLEMENTARY MATERIALS AND METHODS

Cell cycle analysis

U2OS and siRNA-treated HMGB1 depleted U2OS cells were grown to 50-70% confluence in a 60 mm culture dish. Cells were collected by Trypsin-EDTA treatment and washed with cold 1x PBS twice and then were fixed with 70% ethanol for 30 minutes at 4°C. Subsequently cells were treated with ribonuclease and stained with propidium iodide (PI) and analyzed using BDFACS AriaII (BD Biosciences) to analyze the cell populations.

Cell culture, siRNA-mediated HMGB1 depletion and mutagenesis assay

The TP53 null human osteosarcoma SaOS-2 cell line was purchased from ATCC and were cultured in DMEM containing 15% FBS with 5% CO_2 at 37°C. HMGB1 depletion was achieved by treating the cells with GeneSilencer (Genlantis Inc.) transection reagent according to the manufacturer's suggested protocol. Depletion of HMGB1 was confirmed by Western blotting. Mutagenesis assays were performed as described in the main text.

SUPPLEMENTARY FIGURES

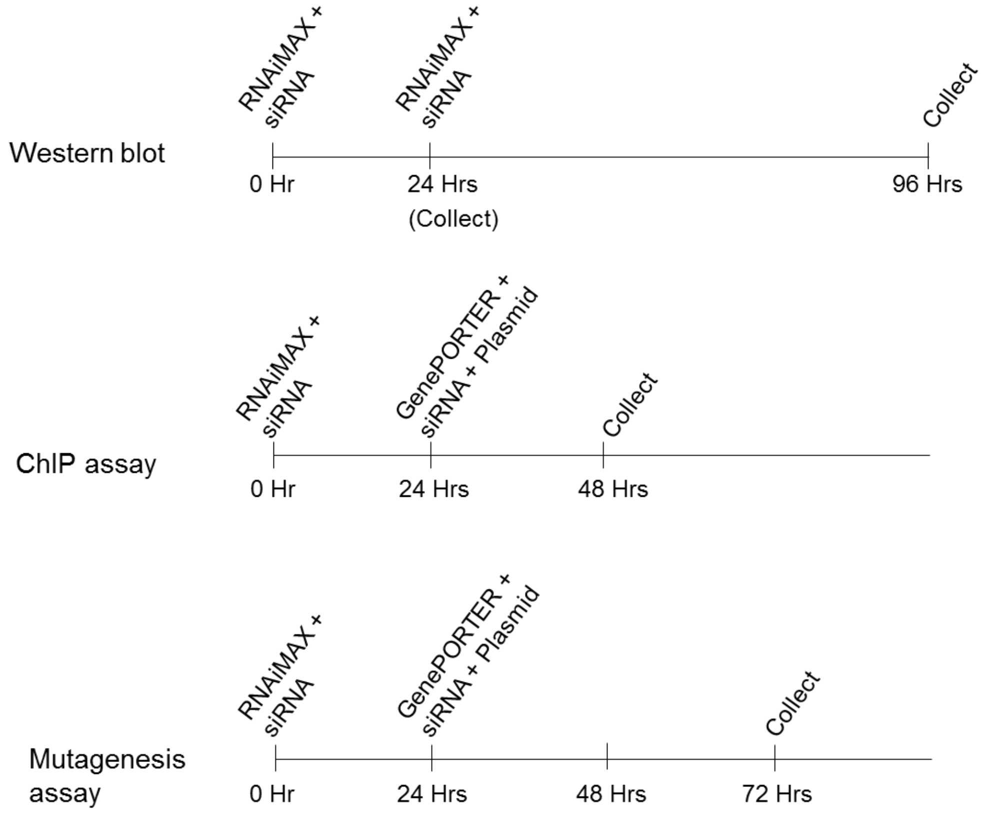

Supplementary Figure 1. Schematic representation of the timeline and reagents used for siRNA and plasmid transfections in human cells for Western blotting, ChIP, and mutagenesis assays. To determine the efficiency of siRNA-mediated HMGB1 depletion by Western blotting, cells were collected at 24 hours and 96 hours. For ChIP and mutagenesis assays in human cells, siRNA was first transfected using RNAiMAX at 0 hour. Plasmids were transfected along with siRNA using GenePORTER as a transfection reagent at 24 hours. For ChIP assays, the plasmids were collected 24 hours after transfection with siRNA+Plasmid, whereas for

mutagenesis assays the plasmids were collected 48 hours after transfection with siRNA+Plasmid.

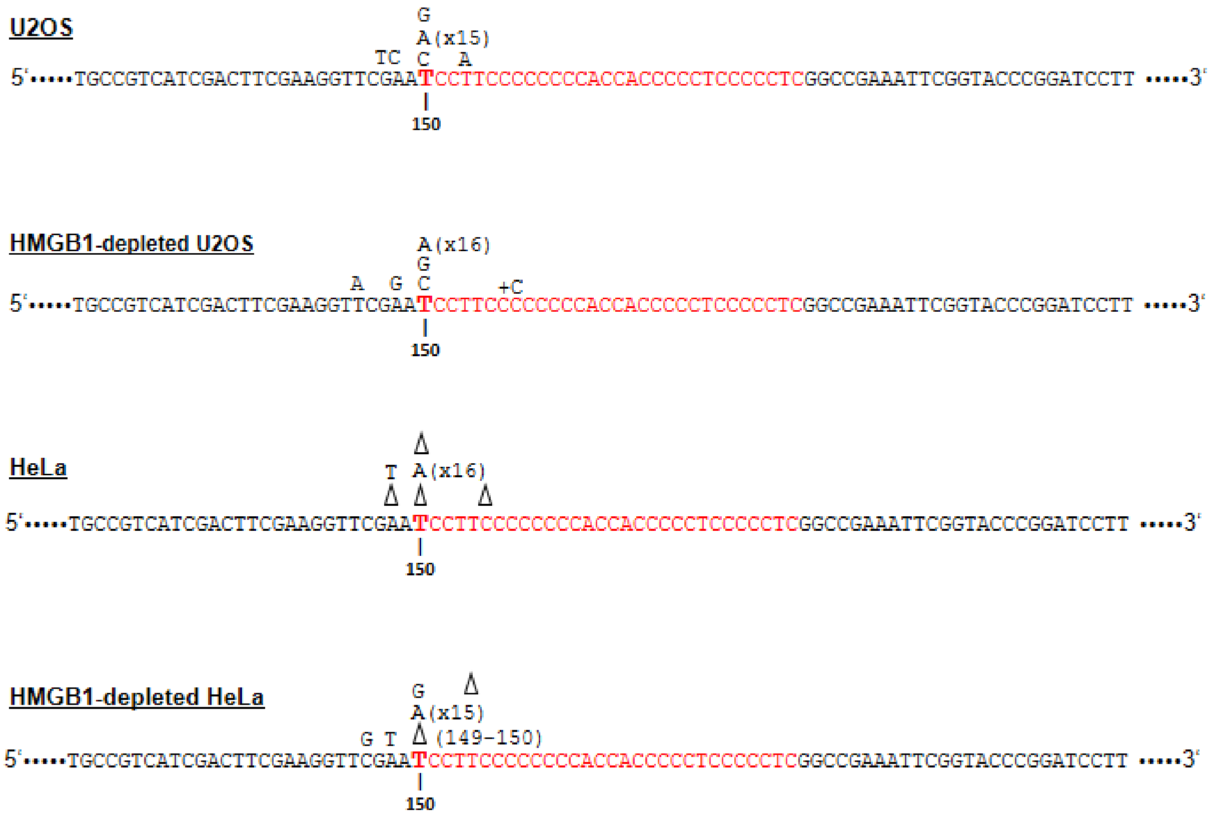

Supplementary Figure 2. ICL-induced mutation spectra from mutant plasmids generated in HMGB1-depleted U2OS and HeLa cells. The TFO-binding site is indicated in red. The base substitutions are indicated above the original sequence at the respective positions. The deletions are indicated with triangles. The TFO-targeted ICL-forming thymine (T) is identified as residue 150. The numbers within parenthesis indicate the number of times a mutation occurred in that specific residue or the region.

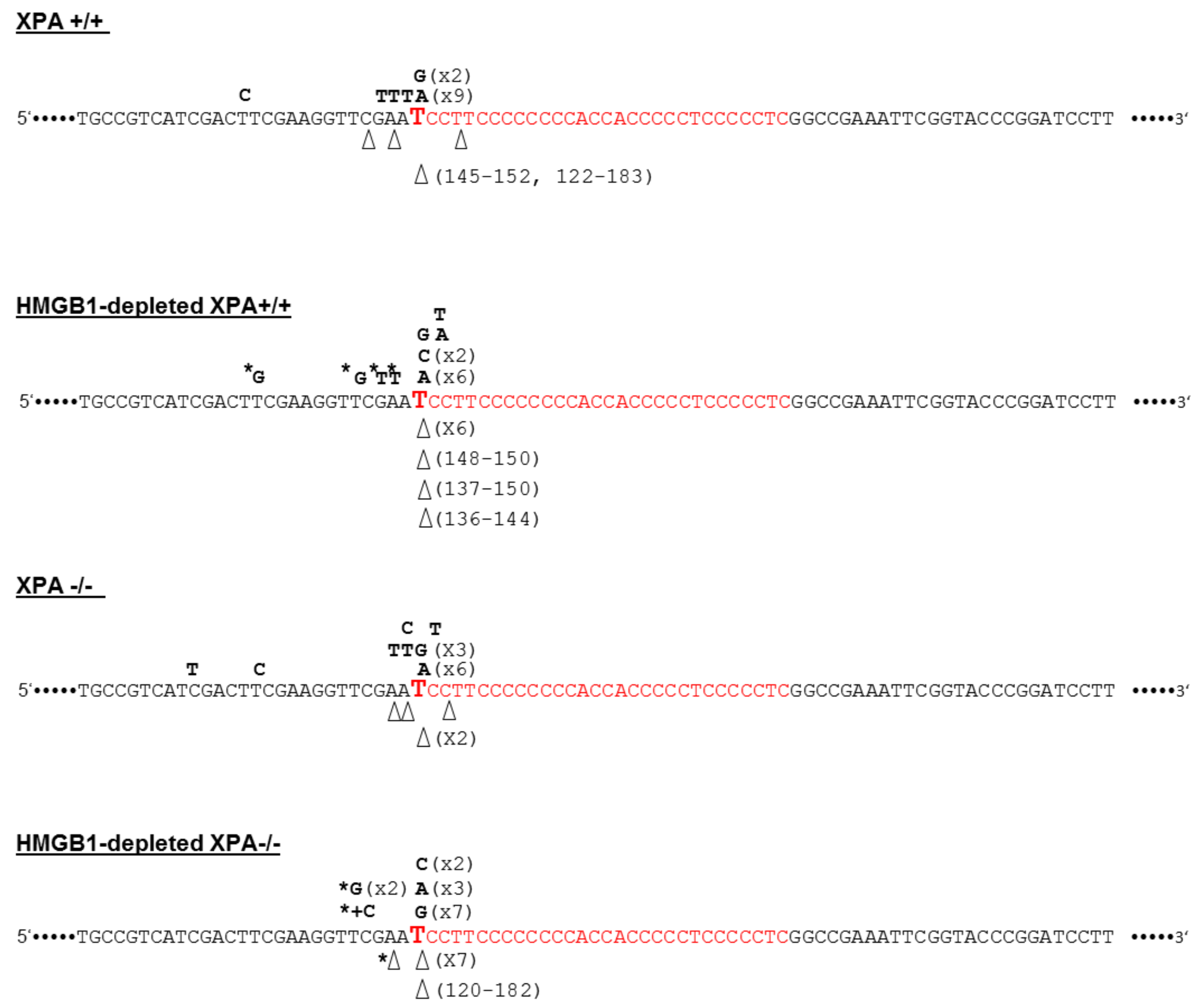

Supplementary Figure 3. ICL-induced mutation spectra of mutant plasmids generated from HMGB1-depleted, XPA-proficient and XPA-deficient cells. The TFO-binding site is indicated in red. The base substitutions are indicated above the original sequence at the respective positions. The deletions are indicated with triangles. The TFO-targeted ICL-forming thymine (T) is identified in larger red bold font. The numbers within parentheses indicate the number of times a mutation occurred in that specific residue or region. Additional mutations in clones have been identified by an asterisk.

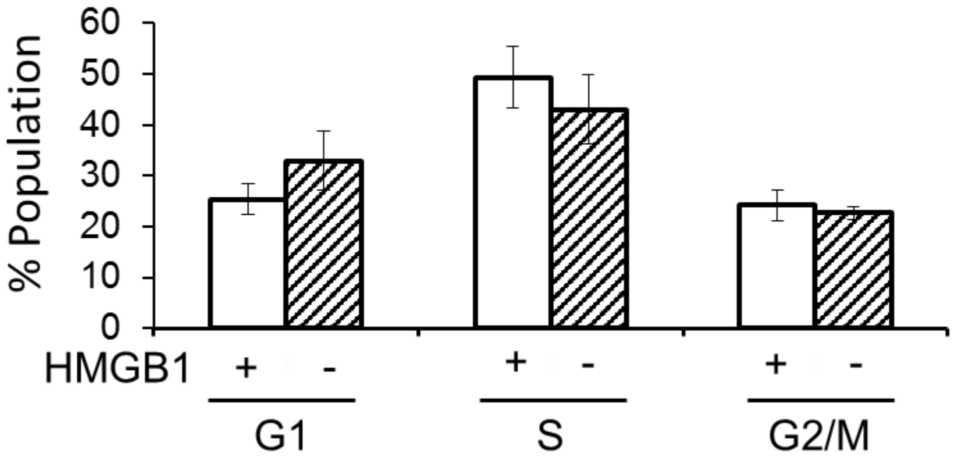

Supplementary Figure 4. HMGB1 depletion does not significantly alter the cell cycle progression in U2OS cells as indicated by cell sorting analysis.

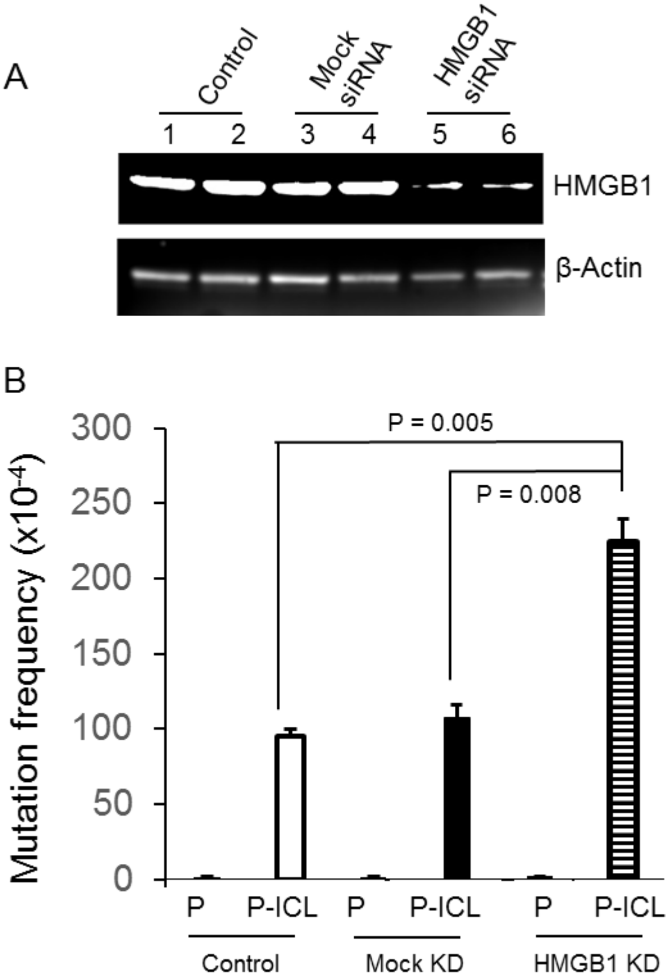

Supplementary Figure 5. HMGB1 depletion in human SaOS-2 cells increases psoralen ICL-induced mutagenesis in a TP53-independent fashion. A. siRNA-mediated depletion of HMGB1 in SaOS-2 (TP53 null) osteosarcoma cells was assessed by Western blotting. Lanes 1 and 2, untreated control; lanes 3 and 4, mock-siRNA treated; lanes 5 and 6, HMGB1-siRNA treated. **B.** Spontaneous (P) and ICL-induced (P-ICL) mutation frequencies in HMGB1-depleted SaOS-2 cells. Cells were transfected with pSupFG1 plasmid (P) or with TFO-directed psoralen-crosslinked pSupFG1 (P-ICL), as indicated under the bars. The TFO-directed psoralen ICLs resulted in an ~60-fold induction in the mutation frequency over the spontaneous levels in control and mock siRNA-treated samples, whereas HMGB1 depletion resulted in >100-fold

induction in mutagenesis. The results are an average of 3 independent experiments; error bars represent ± SD. P values were derived from one-way ANOVA (Holm-Sidak method).
